# Collaborating with a social housing provider supports a large cohort study of the health effects of housing conditions

**DOI:** 10.1186/s12889-016-2730-9

**Published:** 2016-02-16

**Authors:** Michael G. Baker, Jane Zhang, Tony Blakely, Julian Crane, Kay Saville-Smith, Philippa Howden-Chapman

**Affiliations:** He Kainga Oranga/Housing and Health Research Programme, Department of Public Health, University of Otago Wellington, Box 7343, Wellington, 6242 New Zealand; Department of Public Health, University of Otago Wellington, Wellington, New Zealand; Centre for Research, Evaluation and Social Assessment, Wellington, New Zealand

**Keywords:** Social housing, Cohort, ethnicity, Socioeconomic, Crowding, Smoking, Data matching, Data linkage, Health outcomes

## Abstract

**Background:**

Despite the importance of adequate, un-crowded housing as a prerequisite for good health, few large cohort studies have explored the health effects of housing conditions. The Social Housing Outcomes Worth (SHOW) Study was established to assess the relationship between housing conditions and health, particularly between household crowding and infectious diseases. This paper reports on the methods and feasibility of using a large administrative housing database for epidemiological research and the characteristics of the social housing population.

**Methods:**

This prospective open cohort study was established in 2003 in collaboration with Housing New Zealand Corporation which provides housing for approximately 5 % of the population. The Study measures health outcomes using linked anonymised hospitalisation and mortality records provided by the New Zealand Ministry of Health.

**Results:**

It was possible to match the majority (96 %) of applicant and tenant household members with their National Health Index (NHI) number allowing linkage to anonymised coded data on their hospitalisations and mortality. By December 2011, the study population consisted of 11,196 applicants and 196,612 tenants. Half were less than 21 years of age. About two-thirds identified as Māori or Pacific ethnicity. Household incomes were low. Of tenant households, 44 % containing one or more smokers compared with 33 % for New Zealand as a whole. Exposure to household crowding, as measured by a deficit of one or more bedrooms, was common for applicants (52 %) and tenants (38 %) compared with New Zealanders as whole (10 %).

**Conclusions:**

This project has shown that an administrative housing database can be used to form a large cohort population and successfully link cohort members to their health records in a way that meets confidentiality and ethical requirements. This study also confirms that social housing tenants are a highly deprived population with relatively low incomes and high levels of exposure to household crowding and environmental tobacco smoke.

## Background

In New Zealand infectious diseases emerged as an increasing public health problem during the 1990s. Hospitalisation rates from infectious diseases increased by about 50 % during that decade [1]. New Zealand experienced a severe and prolonged meningococcal disease epidemic, which began in 1991 and resulted in disease rates that were about 10 times higher than pre-epidemic levels [2]. In addition, New Zealand has relatively high rates of several diseases spread by respiratory routes and close physical contact, notably rheumatic fever [3] and childhood pneumonia [4].

A case–control study of meningococcal disease conducted in Auckland from 1997 to 1999 showed the risk of disease in children was highly associated with household crowding, as measured by the number of adults and adolescents per room [5]. Other New Zealand studies identified an association between household crowding and the risk of rheumatic fever [6], tuberculosis [7] and childhood pneumonia [4].

Despite the intuitive logic that household crowding contributes to the spread of infectious diseases there is a surprisingly small published literature on the health effects of this exposure and few published studies on the effects of housing interventions to reduce household crowding [8]. A New Zealand review of the effects of crowding on health concluded, “The debate about the relationship between crowding and health is long standing and inconclusive” [9].

The Housing and Health Research Programme (He Kainga Oranga) was formed in response to concerns about the health consequences of poor housing and how these could be addressed. One of its core projects, the Social Housing Outcomes Worth (SHOW) Study, was established to investigate the health effects of housing conditions, particularly household crowding [10]. The study was actively supported by the Chief Executive of New Zealand’s social housing provider, Housing New Zealand Corporation (HNZC) [11]. We know of no other studies that have used large administrative housing datasets to explore household crowding and health issues in this way.

The SHOW Study has the following aims: (1) To describe the characteristics of social housing applicants and tenants and their use of social housing over time; (2) To assess the relationship between household crowding and other household exposures and health outcomes; (3) To assess the impact of placement in social housing on the health status of tenants; (4) To assess the impact of housing improvements on the health of tenants.

Here we report on the methods and feasibility of this study and the first of its aims to describe the characteristics of social housing applicants and tenants.

## Methods

### Design

This research uses a prospective open cohort design to follow a defined population of people over time to measure the relationship between their housing conditions (exposures) and hospitalisations and mortality (outcomes). Planning for this study began in July 2001 with data collection starting in February 2003.

This study was made possible by the support of New Zealand’s largest provider of social housing, Housing New Zealand Corporation (HNZC). HNZC provides housing for approximately 5 % of the population of New Zealand and in the process collects a considerable amount of information to assess the housing needs of applicants and respond to the changing circumstances of tenants.

Social housing represents a relatively small proportion of housing in New Zealand compared to similar European welfare states. Consequently, it has become the housing option of last resort, with tenants generally being amongst the most socio-economically deprived groups in New Zealand.

To meet its aims, this study collected data on key ‘contextual’ factors (notably household exposures) as well as ‘compositional’ aspects of the housed population (notably socio-demographic characteristics) along with linked health outcome data (Table 1).

**Table 1:** Main variables being measured as part of the SHOW Study

Level	Factors	Variables measured
Household & neighbourhood exposures	Environment exposures and access to services	• Property location recorded at small area level allows them to be assigned to region and neighbourhood level exposures and distance to facilities and services
	Household socio-economic status	• Deprivation rating (NZDep) of neighbourhood
	Household crowding level	• Household occupancy and bedroom deficit calculated from information on composition of the household and numbers of bedrooms
	Type of tenure and duration of tenancy	• Housing applicant or tenant •Duration of tenancy
	Household income	• Equivalised household income (generally low compared to NZ population) • Receipt of means-tested Government benefit
	Household type	• Composition of family unit e.g. single person, sole parent, parents and children
	Passive smoke exposure	• Assigned to tenant households based on self-reported smoking behaviour of adult occupants
	Participation in Healthy Housing Programme	• Whether offered programme and acceptance •Nature of specific interventions(s) and timing
Individual level factors	Age	• Date of birth recorded for all participants
	Sex	• Sex recorded for all participants
	Ethnicity	• Self-identified ethnicity recorded for all participants
	Socioeconomic status	• Not specifically measured for individuals (generally low because of the HNZC housing allocation system)
	Established chronic disease and disability	• Not specifically measured for individuals (but has high prevalence because of the HNZC housing allocation system)
	Active smoking	• Assigned to individuals based on self-reported smoking behaviour
Linked outcome data	Hospitalisations	• Diagnostic codes, E codes, timing of admissions, outcomes
	Deaths	• Cause of death, E codes, timing of deaths
	Other outcomes linked to individual NHI	• Pharmaceuticals and other health events linked to individual NHI

### Exposure assessment

The study utilises the fact that HNZC obtains and stores detailed records on all applicants and existing tenants (Table 1). Information on housing applicants is collected via a Needs Assessment (NA) semi-structured interview completed at the time of application for public housing. This NA interview is only conducted on applicants who meet certain eligibility criteria and who are then placed on the waiting list. Information on housing tenants comes from a self-completed Income-Related Rent (IRR) form that is filled out by almost all tenant households each year, or more often if their circumstances change. This information allows HNZC to set the house rental at a level no greater than one-quarter of the household income, which is almost invariably at a lower level than market rental, so there is a strong incentive for households to complete this form. In addition, HNZC records when tenants exit a house, leave the waiting list, transfer from one house to another, or report a change in their circumstances.

These administrative processes allow for collection of key demographic variables (age, sex, ethnicity), crowding (number of people, number of bedrooms) and confounders (household income). A voluntary smoking question was added to the IRR form for completion by all adult household members (those 18 years of age and above). The question is based on that used in New Zealand census questionnaires and reads: “Do you smoke cigarettes regularly (one or more a day)?”

Household crowding can be measured in a number of ways. The simplest is household occupancy, which is the number of people per house. Density measures consider the number of people in relation to the size of the house, for example people per room or bedroom. Crowding measures consider the density according to a defined standard. An example is the Canadian National Occupancy Standard (CNOS), which defines household crowding as a situation where one or more additional bedrooms are required to meet the sleeping needs of the household [12]. Statistics New Zealand has adopted this standard in recent reports [13] as have other agencies, including the Australian Bureau of Statistics [14] and HNZC.

### Outcome measurement

**Fig. 1:**
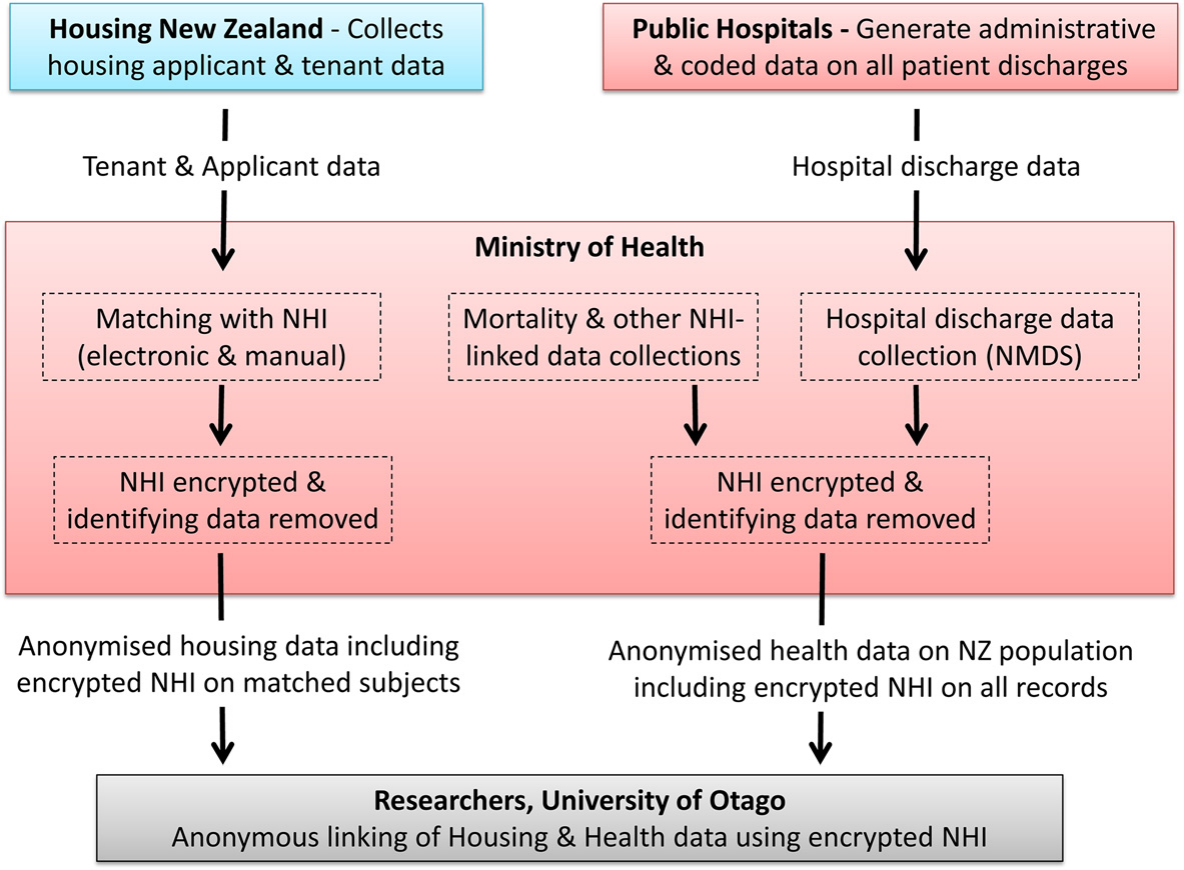
Process of SHOW Study data linkage combining housing data from Housing New Zealand and health data (notably hospital discharge and mortality data) from the Ministry of Health linked using a unique health identifier (encrypted NHI)

This research uses, as the measure of health outcomes, hospitalisations and mortality recorded by the New Zealand Ministry of Health (MoH). The MoH obtains coded data on all publicly funded hospital admissions and deaths in New Zealand (Fig. 1). Each event includes a health sector identifier, the National Health Index (NHI) number, which corresponds to a unique individual resident.

Data on housing applicants and tenants are transferred from HNZC to the MoH for matching with the individual’s NHI (Fig. 1). The MoH uses four data fields (first given name, surname, sex, and date of birth) for matching purposes, with electronic matching, followed by manual matching. The MoH supplies the researchers with the HNZC file along with the NHI for each cohort member where this could be identified. To ensure confidentiality, the names of participants are removed and the NHI in encrypted.

The MoH has also supplied the researchers with a file of all hospital discharges and deaths for New Zealand with the encrypted NHI for each record (Fig. 1). The encrypted NHI enables the researchers to anonymously link cohort members to any hospitalisations or deaths occurring during the study period. The hospitalisation data include diagnosis (up to 20 ICD-10 codes for each event), Ecodes (for injuries), date of admission and discharge, outcome details and administrative data reported by hospitals, such as whether the admission is ‘acute’, ‘arranged’ or from the ‘waiting list’. Mortality data include underlying cause of death coded with ICD-10 and date of death.

### Analysis

The investigators generate the study dataset by merging the anonymised tenant/application data with the anonymised housing data using the encrypted NHI (Fig. 1). This study dataset contains anonymised applicant and tenant data along with details of hospital admissions and deaths that participants had during the study period.

The analysis reported in this paper has focussed on assessing the feasibility of the study and describing the characteristics of the study population. Key indicators of feasibility are the level of data matching achieved and the completeness of key data fields. The characteristics of the cohort study population include its size and dynamic movements over time and its socio-demographic characteristics and levels of household crowding.

Further analysis of the cohort study will use a range of outcome (dependent) variables based on specific diseases (e.g. rheumatic fever, bronchiolitis) and categories of diseases (e.g. skin infections, acute infections generally) as well as non-infectious diseases and injuries.

### Ethics and communication

The study proposal and associated consent forms were supplied to all 12 New Zealand Ethics Committees. After some discussion, the study was approved under the agreed multi-centre process coordinated by the Wellington Health Ethics Committee.

An important step in setting up the study was to inform HNZC housing applicants and tenants that the study was being carried out using their records but that they would not be identifiable to the researchers. Information about the study is included in a privacy statement on the NA and IRR forms. A separate pamphlet about the study was included with NA interview and IRR forms. Versions of this pamphlet were prepared in other languages (Māori, Pacific languages and Chinese) and made available as required. A free phone information number was set up and staffed by University of Otago staff for HNZC clients who wanted more information about the study. Information was also included in HNZC tenant newsletters and press releases. HNZC tenancy managers received information packs about the study and training material for use with their staff.

## Results

### Data matching and exclusions

During the 8-year period Jan 2004 to December 2011 the MoH matched approximately 96 % of subjects to their unique NHI numbers. The initial electronic matching was 66 % with the remainder by manual matching. Consequently, some applicants (3.6 %) and tenants (4.3 %) were excluded from the study. A small number of tenants (2.7 %) were not included in the study because they did not apply for subsidised income-related rent (IRR).

### Completeness of information

Analysis of HNZC data shows a high degree of completeness for the fields of interest, including date of birth (100 %), sex (100 %), ethnicity (99 % for Needs Assessment and 98 % for IRR) averaged across 8 years. These are required fields in the HNZC tenancy management system so this result was not surprising, but it does illustrate the benefits of using administrative data. Response to the voluntary smoking question added to the IRR was considerably lower, averaging 70 % during the course of the study.

### Number of households in the cohort

**Fig. 2:**
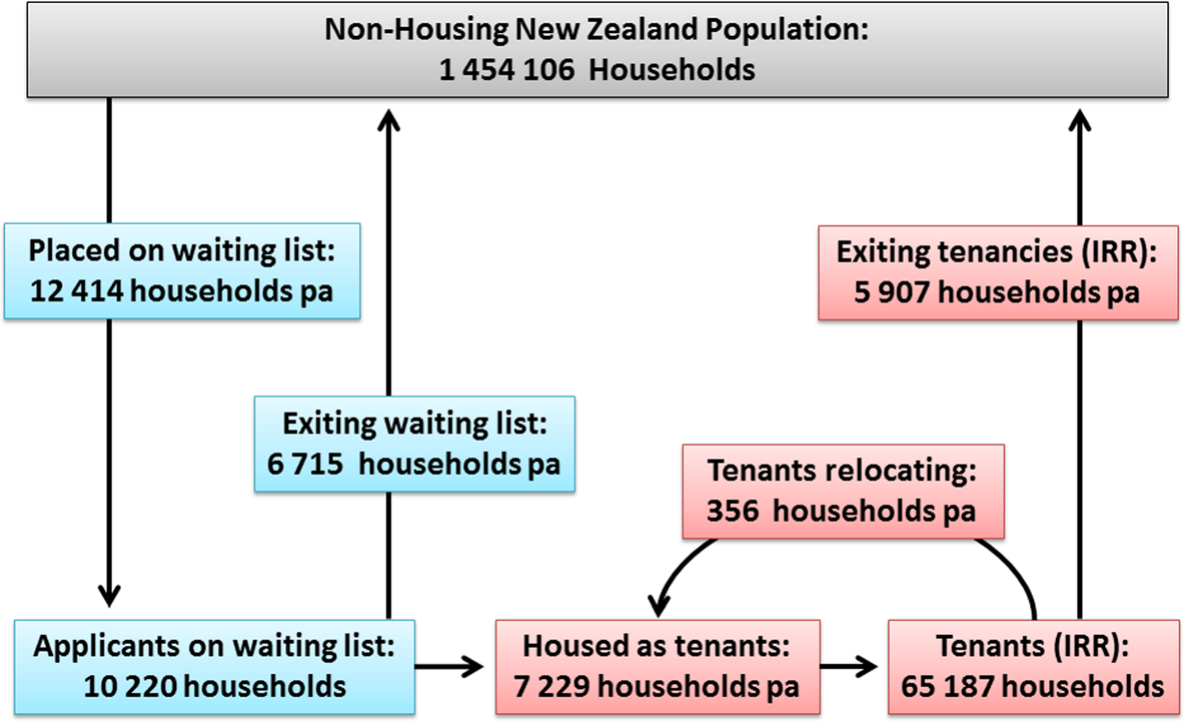
Numbers of households moving into and out of the SHOW Study populations (Applicants on waiting list and Tenants with IRR) showing average numbers of households in each population and average annual flows between these populations and the wider New Zealand population over the 8-year period 2004–11

Figure 2 shows the average number of households included as social housing applicants on the waiting list (10,220 households, 24,608 people) and as tenants receiving IRR (65,187 households, 197,317 people) during the 8-year period 2004–2011. As this flow-chart illustrates, the population is a dynamic one with an average of 12, 414 new applicant households (29,518 people) placed on the waiting list and 7229 households (17,189 people) housed in HNZC properties each year over that period. A cross-section of applicants and tenants can be constructed at any point in time by pooling those who have entered the applicant and tenant populations at that point, and deducting those who are known to have exited.

### Characteristics of the study population

This analysis was conducted on a cross-section of housing applicants and tenants as at 31 December 2011. Important characteristics of the housing applicant and tenant population are summarised in Table 2.

**Table 2:** Characteristics of SHOW Study participants (applicant and tenant households at Dec 2011) compared with the New Zealand population (2006 census and 2013 census)

Characteristic	Housing applicants^a^	Housing tenants^b^	NZ population^c^ (2006)	NZ population^d^ (2013)
Population				
Number of households	4931	64,706	1,454,106	1,570,695
Number of people	11,196	196,612	4,027,947	4,242,048
Median duration on waiting list (*current* applicants)	34 weeks	–	–	
Median duration in tenancy	–	212 weeks	–	
Demographic and socio-economic characteristics				
Age and sex				
Median age	21	21	36	38
Female %	55.5	54.8	51.2	51.3
Ethnicity^d^				
European %	25.5	27.6	64.7	70.0
New Zealander %	–	–	10.7	1.6
Māori %	32.6	38.5	14.0	14.1
Pacific %	34.0	39.4	6.6	7.0
Asian %	10.6	4.4	8.8	11.1
Middle Eastern Latin American %	7.1	3.1	0.9	1.1
Other %	0.6	0.4	0.04	0.04
Not Stated %	1.4	1.7	4.3	5.4
Economic indicators				
One parent with children %	35.7	35.4	18.1	17.8
Average weekly household income $	356.9	387.5	1321.6^e^	1398.4
Receipt of income from Gov. benefit %	86.8	95.6	24.5	25.1
Smoking status^f^				
Smoker in household %	–	44.1	32.9	
Proportion of adults who smoke %	–	29.9 (18+ years)	20.7 (15+ years)	15.1 (15+ years)
Crowding levels				
Sharing with another family %	41.5		2.8	3.3
Average number of people in household	4.2	3.2	2.7	2.7
Average number of bedrooms	2.5	2.6	3.1	3.1
Average people per bedroom	1.7	1.2	0.9	–
Short of 1 or more bedrooms %				
Household	44.3	22.3	5.1	
Person	52.3	38.0	10.0	
Short of 2 or more bedrooms %				
Household	22.2	7.1	1.2	
Person	28.3	14.3	3.5	–

^a^Housing applicants are those who have been “confirmed” and placed on the waiting list for a house

^b^Housing tenants are those who complete an Income Related Rent application form. This group excludes 1750 HNZC tenant households not claiming this benefit (i.e. who are paying market rent)

^c^Based on 2006 NZ Census

^d^Ethnic Group. This is based on grouped total responses. Where a person reported more than one ethnic group, they have been counted in each applicable group

^e^From New Zealand income survey at June 2006

^f^Based on the tenants (69 %) who reported their smoking status

This analysis shows that housing applicants and tenants are a young population compared with the total New Zealand population, with half less than 21 years of age. Māori and Pacific people make up 59 % of applicant and 74 % of tenant households. Single parents with children make up 36 % of the applicant households and 35 % of the tenant households. Household incomes are comparatively very low for applicant and tenant households. The majority of applicants (87 %) and tenants (96 %) received income from a Government benefit. A higher proportion of adult housing tenants smoke (30 %) than for New Zealanders as a whole (21 % in the 2006 census and 15 % in the 2013 census). Similarly, a higher proportion of tenant households (44 %) compared with New Zealand as a whole (33 %), were potentially exposed to passive smoking, based on the households containing one or more smokers.

### Crowding levels of study population

**Fig. 3:**
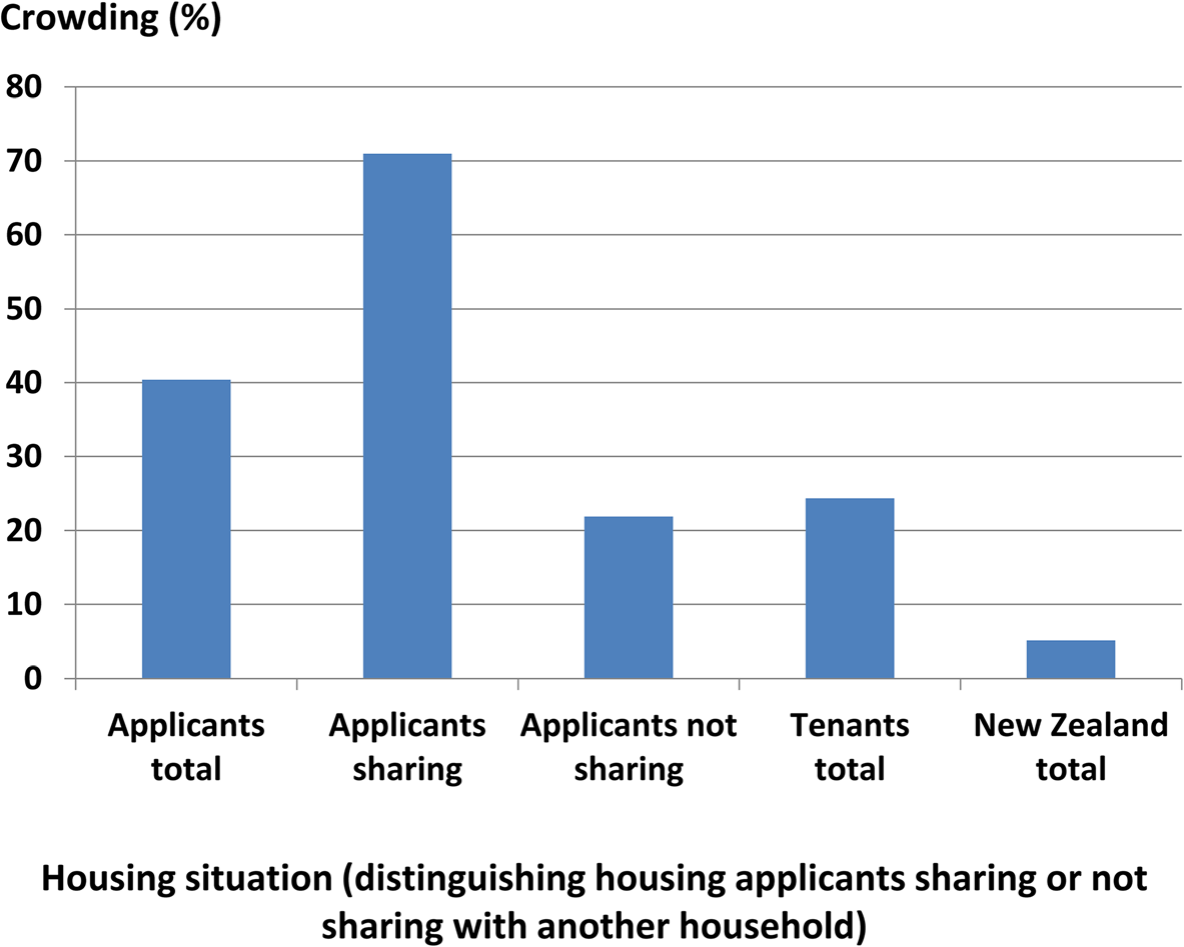
Household crowding levels of social housing applicants and tenants compared with the total New Zealand population. Crowding is measured using the Canadian National Occupancy Standard (CNOS), which considers a household to be crowded where it is short of one or more bedrooms for its occupants. The crowding level is the percentage of each group which has a bedroom deficit of one or more

About 10 % of New Zealanders are exposed to household crowding, as measured by a deficit of one or more bedrooms in 2006 [15]. By contrast, about 44 % of housing applicants and 22 % of tenants live in households with this level of household crowding (Fig. 3). A particularly exposed group are the 41 % of housing applicants living with other families. They reported high levels of household crowding (72 % of these ‘double-up’ households were classified as crowded using the CNOS, compared with 25 % of applicant households who were not sharing).

This initial analysis has also investigated changes in household crowding levels that occur when housing applicants become HNZC tenants. During the 8-year observation period, an average of 7229 households a year went from being applicants to tenants. This population included 2941 households a year known to have household crowding (1+ bedroom deficit), with the majority (an average 94 %) having a reduction in crowding after moving to a HNZC house. This group is important for the investigation of one of the main hypotheses of the study that a decrease in crowding level is associated with a drop in disease risk.

## Discussion

This research has demonstrated that it is possible to establish a large cohort study using administrative data collected by a public housing provider. It has also shown that a high proportion of subjects in this database can be linked to their health records in a way that is ethically acceptable and protects privacy. This population of social housing applicants and tenants is characterised by its young age, high proportion of Māori and Pacific peoples, and sole parent households. It has a low income and high level of receipt of Government income support.

These results also show that this population is highly exposed to household crowding and household smoking, compared with the New Zealand population more generally. It has also demonstrated that the majority of housing applicants who became tenants decrease their level of household crowding in the process. This study therefore has the potential to add to the relatively small evidence base of research on the health effects of housing conditions. A further advantage of this study is that by focussing on social housing tenants, who are a particularly vulnerable population, it partly redresses the balance in other New Zealand cohorts which are disproportionately European.

Using administrative databases, such as that collected by HNZC, has the advantage of providing a potentially large cohort population at relatively low cost. Similarly, using established national hospitalisation and mortality databases to identify health outcomes in this cohort allows a far larger study size than would otherwise by possible. This study design also lets the researchers investigate multiple health outcomes potentially allowing the study to quantify a larger proportion of the overall burden of disease that can be attributed to household conditions than could be done with individual disease studies. It also enables researchers to analyse the effect of important changes to social housing policy that have occurred during the course of the cohort.

This study design and methods have a number of potential weaknesses and limitations that are common to many observational epidemiological studies, notably confounding, selection bias, information bias, and issues of generalisability.

The high level of matching of cohort members with health data is important for minimising potential selection bias. Such a bias could occur if, for example, the relationship between crowding and disease risk was different for those included (matched) compared with those who were not included (unmatched) [16]. Given the high proportion that is matched, this bias is unlikely to be important.

Household crowding is highly associated with other measures of socio-economic deprivation such as low income, unemployment, low education level and fewer material resources. Other risk factors for respiratory disease, notably active and passive smoking, are also more prevalent in crowded households [17]. These confounders could have the effect of producing or increasing the measured cross-sectional association between household crowding and increased hospitalisations. Several potential confounders, notably age, sex, and ethnicity are well recorded and will be used in the analysis. Because the study population is defined on socio-economic grounds (as particularly deprived) some confounders are effectively controlled by restriction. Data on active and passive smoking are also recorded for more than half of the households, which will permit analysis of this effect on a substantial sub-group. But most importantly, the longitudinal component of this study, which follows participants as they change their levels of crowding over time, will effectively overcome the issue of confounding by other important and relatively fixed covariates, such as chronic disease and disability status.

Some study data are dependent on the accuracy of information that housing applicants and tenants supply to HNZC, which introduces potential for information bias of exposures and covariates. This particularly applies to information on the number of people living in the applicant and tenant households. Housing applicants may tend to over-state the number of people in their homes to increase their priority on the waiting list. Housing tenants may under-count the number of people staying with them to minimise their rent (though HNZC tenants can house two boarders without it counting as income for the calculation of income-related rent). Consequently, if there truly was an association of reduced hospitalization rates due to reduced household crowding after placement from an applicant to tenanted house, we may only detect such a reduction for apparent reductions in household crowding that exceeded those purely due to biased recording. This effect will be considered in a sensitivity analysis of the findings.

Because this study is restricted to social housing users, who are by definition a socio-economically deprived group in New Zealand, the generalisability of the findings to the total population may be limited. However, as noted previously, a major aim of this cohort is to track the impact of the move to social housing for this economically-deprived population rather than for the New Zealand population in general. Because of the cohort design, this study will also allow researchers to see whether the role of household crowding that has so far been mainly seen for infectious diseases in children, can be generalised to a wider range of infectious and non-infectious diseases where such a role seems plausible.

## Conclusions

The SHOW Study has demonstrated that an administrative housing database can be used to form a large cohort population and successfully link cohort members to their health records in a way that meets confidentiality and ethical requirements. It confirms that social housing tenants are a highly deprived population with relatively low incomes and high levels of exposure to household crowding and environmental tobacco smoke.

This study provides a mechanism for investigating the role of housing conditions, such as household crowding, as a risk factor for a range of diseases and injuries. By taking advantage of a ‘natural experiment’ where large numbers of housing applicants are re-housed in public housing, this study could also add to the small literature on the health effects of housing improvements [8].

This study also demonstrates the value of partnerships between researchers and service providers. By building research and evaluation into service deliver processes it is sometimes possible to provide research findings in a more efficient way than with ‘stand-alone’ research projects. Now that it is established, this study could be extended at relatively low cost to investigate other links between housing conditions and health outcomes. Such mechanisms provide benefits for the service provider as well as the research and policy end-users.
